# Laparoscopic surgery for endometrial cancer is oncologically safe and improves hospital stay duration: a retrospective single-center study over a 16-year period

**DOI:** 10.1007/s00404-024-07550-x

**Published:** 2024-05-25

**Authors:** Anna-Christina Rambow, Moritz Nikolai, Peer Jansen, Christoph Rogmans, Nils Tribian, Dirk O. Bauerschlag, Nicolai Maass, Marion T. van Mackelenbergh

**Affiliations:** Department of Gynecology and Obstetrics, University Hospital Kiel, Arnold-Heller-Straße 3, 24105 Kiel, Germany

**Keywords:** Endometrial cancer, Total laparoscopic hysterectomy, Total abdominal hysterectomy, Lymphonodectomy, Overall survival

## Abstract

**Objective:**

To investigate changes in surgical procedures and patient outcomes of patients diagnosed with endometrial cancer (EC) at a German university hospital between 1998 and 2014.

**Methods:**

A monocentric, retrospective review was conducted to identify patients diagnosed and treated with EC during the aforementioned period at the Department of Gynecology and Obstetrics at the University Hospital Kiel, Germany.

**Results:**

303 patients were identified. Patient demographics, risk factors, histological subtypes and stages of EC remained consistent over time. The most common surgical procedure was total abdominal hysterectomy (TAH) (81.9%). In 2011, the institution carried out its first total laparoscopic hysterectomy (TLH) for EC, resulting in a significant increase in laparoscopic surgical procedures (2011–2014: *N* = 70; TAH 44.2%; TLH 51.4%). Although the total number of lymph node stagings remained consistent over time, there was a significant increase in the performance of simultaneous pelvic and para-aortic lymphonodectomy (LNE) compared to pelvic LNE alone (2.6 in 2001–2005 vs. 18.0% in 2011–2014, *p* ≤ 0.001). The duration of hospital stays significantly decreased over time, with a mean of 20.9 days in the first and 8.5 days in the last period. When comparing surgical procedures, TLHs resulted in significantly shorter postoperative stays with an average of 6.58 vs. 13.92 days for TAH. The surgical procedure performed did not affect 5-year overall survival rates in this study (84.9% for TAH and 85.3% for TLH, *p* = 0.85).

**Conclusions:**

Our retrospective single-center study demonstrates that laparoscopic surgery for endometrial cancer is oncologically safe and shortens hospital stays.

## What does this study adds to the clinical work

**Table:** 

Total laparoscopic hysterectomy is oncologically safe for endometrial cancer. Laparoscopic surgery for endometrial cancer shortens hospital stay duration.	

## Introduction

Endometrial cancer (EC) is a prevalent gynecologic cancer worldwide. The incidence of EC varies by region, with the highest rates observed in Northern America and Western European countries. In Germany, EC is the fourth most common malignancy in women, with almost 11,000 new cases reported annually. The lifetime risk for German women is 2.1%, and the mean age at primary diagnosis is 67 years [1].

Known risk factors for endometrial cancer include advanced age, obesity, diabetes mellitus, hormonal influences (including tamoxifen therapy), and other malignancies such as breast cancer in personal history or hereditary predispositions like HNPCC [2].

EC is typically diagnosed in early stages (stage I) due to symptoms such as postmenopausal bleeding or bleeding disorders in the premenopause, in combination with a suspicious endometrium in vaginal ultrasound. However, some women still experience advanced stages of the condition, requiring extensive surgery such as radical hysterectomy, lymphonodectomy (LNE), and adjuvant therapies comprising radiation, chemotherapy, and/or endocrine therapy.

The standard surgical treatment for EC is total hysterectomy and bilateral salpingo-oophorectomy, which can be performed either laparotomically or laparoscopically. Currently, laparoscopic surgical techniques are the preferred method for treating EC [3]. Most randomized controlled trials (RCTs) that compare open surgery with minimal invasive surgery have found equivalent oncologic safety for both techniques [4]. However, it is important to note that the majority of these studies focus on early-stage EC, while trials including higher stage EC are rare and have low case numbers [5–8].

In addition to equivalent disease-free survival (DFS) and overall survival (OS), some studies have reported beneficial secondary endpoints for laparoscopic surgery, including reduced intra- and postoperative morbidity and shorter hospital stays [9–11].

The objective of this study was to quantify changes in patient characteristics and the management of all stages of EC between 1998 and 2014 at a single university hospital in Germany. The study focused on surgical treatment, duration of hospital stay, and patient outcomes.

## Materials and methods

This observational retrospective monocenter study was conducted at the Department of Gynecology and Obstetrics of the University Hospital Schleswig–Holstein, Campus Kiel. The study included patients diagnosed with EC between January 1998 and December 2014, who had given informed consent for the use of their specimen and clinical data for research purposes.

303 patients with EC were included in the study. The patients were divided into cohorts based on the year of diagnosis: 1998–2000, 2001–2005, 2006–2010, and 2011–2014. The last interval was selected to indicate the implementation of the first total laparoscopic hysterectomy (TLH) for EC at the institution in 2011. The study collected data on patient characteristics and risk factors, including age, menopausal status, BMI, diabetes mellitus, suspect vaginal bleeding, and suspect endometrium, as well as pathology and operation reports from the institution’s electronic data processing system. The patients who underwent therapy until 2010 were staged according to the 1989 FIGO classification system [12], with the revised FIGO staging system being applied from the beginning of 2010. The pathology reports of patients who underwent surgical treatment between 1998 and 2010 were restaged based on the 2009 FIGO staging system [13], using the original pathology report. Patients with cervical stromal involvement were classified as stage II, while cervical glandular involvement was classified as stage I disease (previously also known as FIGO II).

Statistical analyses were conducted using the Statistical Package for Social Sciences for Windows (IBM SPSS Inc, Chicago, IL). Descriptive statistics of categorical variables were presented as means, medians, and ranges expressed as numbers and percentages. Comparative analyses of the different time cohorts were performed using appropriate tests such as Chi-Square, Likelihood Ratio, Kruskal–Wallis, Mann–Whitney, and ANOVA for samples with non-normal distributions. Graphs were plotted using GraphPad Prism Version 9 (GraphPad Software Inc, San Diego, CA).

Disease-free survival (DFS) was defined as the time from diagnosis to recurrence of the tumor or death, and overall survival (OS) was defined as the time from diagnosis to death. Patients lost to follow-up were censored. Survival differences were analyzed using Kaplan–Meier estimation. A significance level of *p* < 0.05 was used.

## Results

Between 1998 and 2014, the Department of Gynecology and Obstetrics at the University Hospital Schleswig–Holstein, Campus Kiel treated 303 patients diagnosed with EC. The mean age of the patients was 66.6 years (range 38–90 years), with 44.2% of enrolled patients being older than 70 years and 89.4% being diagnosed postmenopausal. Our investigation focused on the occurrence of obesity and diabetes mellitus in our cohort, as previously described risk factors. Obesity, defined as a body mass index (BMI) of 25 kg/m^2^ or higher, was observed in 67.2% of cases, with a mean BMI of 29.3 ± 7.625 kg/m^2^. Diabetes mellitus was present in 17.2% of cases. Postmenopausal or abnormal premenopausal bleeding was reported in 70.4% of the overall cohort, and a suspect endometrium was observed by vaginal ultrasound in 79.6% of cases. The patients’ characteristics and risk factors remained stable throughout the study period. However, the prevalence of diabetes mellitus increased steadily from 16% in 1998–2000 to 19.2% in 2011–2014 (*p* = 0.975) (see Table 1).

**Table 1:** Patient demographics and clinical characteristics divided by time cohorts

	Total *N* (% valid)	1998–2000	2001–2005	2006–2010	2011–2014
*Age (years)*					
*N*	303	55	76	99	73
<60	83 (27.4)	10 (18.2)	22 (28.9)	29 (29.3)	22 (30.1)
60–69	86 (28.4)	20 (36.4)	19 (25.0)	29 (29.3)	18 (24.7)
≥70	134 (44.2)	25 (45.5)	35 (46.1)	41 (41.4)	33 (45.2)
*Menopausal status*					
Postmenopausal	273 (90.1)	51 (92.7)	67 (88.2)	92 (92.9)	63 (86.2)
Premenopausal	30 (9.9)	4 (7.3)	9 (11.8)	7 (7.1)	10 (13.7)
*BMI (kg/m*^2^)					
<25	91 (32.7)	11 (22.9)	27 (37.0)	30 (32.6)	23 (35.4)
25–30	81 (29.1)	19 (39.6)	22 (30.1)	24 (26.1)	16 (24.6)
30–40	81 (29.1)	14 (29.2)	18 (24.7)	27 (29.3)	22 (33.8)
>40	25 (9.0)	4 (8.3)	6 (8.2)	11 (12.0)	4 (6.4)
*Diabetes mellitus*					
D.M.	52 (17.7)	8 (16.0)	13 (17.3)	17 (17.7)	14 (19.2)
*Symptoms*					
Suspect vag. bleeding	190 (70.4)	35 (77.8)	46 (64.8)	65 (73.0)	44 (67.7)
Suspect Endometrium	215 (79.6)	33 (73.3)	59 (80.8)	69 (77.5)	54 (85.7)

Pathological analyses identified the endometrioid subtype in 256 cases (84.5%), while non-endometrioid subtypes, including serous, clear cell, and other differentiations, accounted for 7.6, 3.6, and 4.3% of cases, respectively (see Table 2). The study found that the distribution of various types of carcinomas, including mucinous, papillary, squamous, and undifferentiated carcinoma, as well as leiomyosarcoma, endometrial stromal sarcoma, and malignant mixed Müllerian tumor, remained consistent over time (*p* = 0.144).

The majority of endometrial cancers were diagnosed in early tumor stages, with FIGO stage Ia and Ib accounting for over 77% of all cases. Higher stages, FIGO II, III, and IV, were found in 8.5, 12.3, and 1.7% of cases, respectively (see Table 2). The original pathological reports from 1998 to 2010 were reviewed and updated to conform to the new FIGO classification system presented in 2010. No changes to this distribution were observed over time. A diagnostic hysteroscopy was performed prior to surgical therapy in 88.7% of cases. This two-stage procedure was common throughout all time periods (*p* = 0.239).

The majority of patients underwent abdominal hysterectomy (AH) (81.9%). Vaginal hysterectomy (VH) was performed in 6.0% of cases, with no significant changes over time. In 2011, the University Hospital Kiel performed its first total laparoscopic hysterectomy  for EC, leading to a significant decrease in the number of AHs performed at the institution. Between 2011 and 2014, 70 surgeries were performed, consisting of 31 AHs (44.2%) and 36 TLHs (51.4%). To avoid the spread of malignant cells, the laparoscopic approach was routinely performed without the working insert of the uterine manipulator, which enters the uterine cavity.

**Fig. 1:**
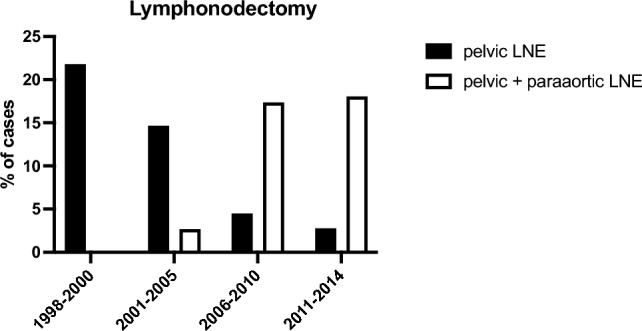
Performance of pelvic and para-aortic lymphonodectomy (LNE) divided by time cohorts

Pelvic lymph node examination (LNE) was conducted in 9.6% of the cases, while combined pelvic and para-aortic LNE were performed in 10.6% of the cases. The first para-aortic LNE was carried out in 2005. During the time periods examined, the performance of combined pelvic and para-aortic lymph node dissection (LND) increased significantly, in contrast to pelvic LND alone (2.6 in 2001–2005, 18.0% in 2011–2014, *p* ≤ 0.001) (Fig. 1). However, the overall performance of LND did not change (21.8 in 2001–2005, 20.8% in 2011–2014). Considering all stages of endometrial cancer, lymph node dissection was performed during AH in 21.8% of the cases and during TLH in 16.2% of the cases. Combined pelvic and para-aortic LNE was performed in 11.1% of AH and 13.5% of TLH. Metastases were found in 25.9% of all LNDs. The percentage of positive lymph nodes increased over time, but this change was not statistically significant (11.1 in 2001–2005; 40% in 2011–2014; *p* = 0.509) (see Table 3).

**Fig. 2:**
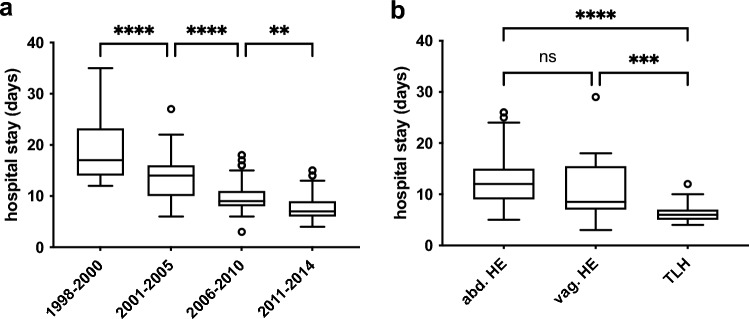
Comparison of hospital stay by **a** time cohorts, **b** surgical procedure

The length of postoperative hospitalization decreased significantly from 20.9 ± 10.37 days in the first time period to 8.5 ± 4.26 days in the last, which means that the time of hospitalization more than halved in the observation period of 16 years. A correlation between hospital stay and surgical procedure was observed, with the longest postoperative stay after AH (13.92 ± 7.68 days) and the shortest after TLH (6.58 ± 2.21 days; *p* ≤ 0.001) (Fig. 2).

Adjuvant treatment decisions were made by a multidisciplinary tumor board, with 52.9% of all cases receiving adjuvant therapy. Of these cases, 41.3% underwent radiation, 8.3% received chemotherapy, and 3.3% were treated with endocrine therapy. While the proportion of chemotherapy and endocrine therapy remained constant over time, the number of patients receiving radiation increased from 27.3% in the first time period to 43.8% in the last time period (*p* = 0.01) (see Table 3).

**Fig. 3:**
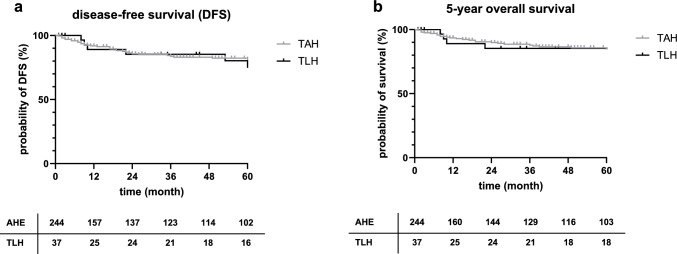
Disease-free survival (DFS) **a** and 5-year overall survival **b** compared by surgical procedure; tables below graphs indicate numbers at risk; *AHE* abdominal hysterectomy, *TLH* total laparoscopic hysterectomy

Follow-up data are available for some patients who underwent TAH or VH up to 21 years after diagnosis of EC. As the first TLH for EC was performed in 2011, follow-up data are available up to 9 years after diagnosis. Overall 5-year survival was 89.29%, overall 10-year survival was 85.98%, overall 15-year survival was 83.76%, and overall 20-year survival was 73.29%. Survival rates did not differ significantly by surgical procedure (*p* = 0.85), with a 5-year overall survival rate of 84.9% for TAH and 85.3% for TLH. In addition, recurrence rates did not vary significantly by surgical procedure (*p* = 0.9). The 5-year recurrence rate was 9.3% for TAH and 15.2% for TLH (Fig. 3). For TAH, the recurrence rates were 25.15% at 10 years, 29.63% at 15 years, and 38.43% at 20 years. VH was excluded from these calculations due to the small number of cases with no documented recurrence or death during the first 5 years after diagnosis (data not shown). Distant metastases were found in the lung, bone, liver, and brain.

## Discussion

This study presents the main trends in surgical treatment of EC at a German university hospital between 1998 and 2014.

Patient and tumor characteristics remained stable over time, but significant changes were observed in surgical approaches and postoperative stay. The institution implemented two milestones in surgical therapy for EC: laparoscopic surgery in 2005 and para-aortic LNE in 2011.

The study demonstrates a significant decrease in postoperative stay duration over time cohorts (20.9 ± 10.37 in 1998–2000 vs. 8.5 ± 4.26 days in 2011–2014, *p* ≤ 0.0001), which is dependent on the surgical procedure. Patients who underwent AH stayed in the hospital for twice as long as those who underwent TLH (13.92 ± 7.68 vs. 6.58 ± 2.21 days; *p* ≤ 0.0001). These findings are consistent with previous literature, which suggests that the laparoscopic approach can reduce hospital stay duration by approximately half. In international comparison, hospital stays in Germany were generally longer than in other countries (cf. Tozzi 2005, Germany: 11.7 after AH vs. 8.6 days after TLH for early-stage EC; Baum, Germany: 12.25 vs. 5.73 days; Fram 2002, Australia: 5.5 vs. 2.3 days; Lu 2013, China: 6 vs. 3 days; Malzoni 2009, Italy: 5.1 vs. 2.1 days; Zorlu 2005, Turkey: 8.2 vs. 4.1 days; Zullo 2009, Italy: 6.9 vs. 3.0 days, Mourits 2010: 5 vs. 2 days; Gao, China: 17.7 vs. 14.7 days) [9, 14–21]. It can be inferred that the changes in operative techniques are not the only contributing factor, but external effects such as differences in hospital payment methods (e.g., diagnostic-related groups (DRGs) in Germany [22]) also play a role .

**Table 2:** Pathological characteristics divided by time cohorts

	Total *N* (% valid)	1998–2000	2001–2005	2006–2010	2011–2014	*p* value
*Histology*						0.144
*N*	303	53	79	99	73	
Endometrioid	256 (84.5)	45 (81.8)	66 (86.8)	84 (84.8)	61 (83.6)	
Serous	23 (7.6)	7 (12.7)	3 (3.9)	10 (10.1)	3 (4.1)	
Clear cell	11 (3.6)	1 (1.8)	5 (6.6)	3 (3.0)	2 (2.7)	
Others	13 (4.3)	2 (3.6)	2 (2.6)	2 (2.0)	7 (9.6)	
*FIGO*						
*N*	294	53	74	99	68	
Ia	179 (60.9)	34 (64.2)	45 (60.8)	64 (64.6)	36 (52.9)	
Ib	49 (16.7)	7 (13.2)	9 (12.2)	14 (14.1)	19 (27.9)	
II	25 (8.5)	4 (7.5)	10 (13.5)	6 (6.1)	5 (7.4)	
IIIa	14 (4.8)	3 (5.7)	5 (6.8)	5 (5.1)	1 (1.5)	
IIIb	3 (1.0)	0 (0)	1 (1.4)	2 (2.0)	0 (0)	
IIIc	19 (6.5)	3 (5.7)	4 (5.4)	5 (5.1)	7 (10.3)	
IV	5 (1.7)	2 (3.8)	0 (0)	3 (3.0)	0 (0)	

The performance of TLH for EC led to a pronounced reduction of the performance of AH while the number of VH did not change (see Table 3). VH was only performed in 18 cases (6%) and exclusively for early stages of EC (FIGO Ia and Ib). Therefore, the OS and DFS curves cannot be compared to those of AH and TLH. No deaths or recurrence were observed over 177 months of follow-up for patients who received VH (data not shown). However, our results for OS and DFS are consistent with previous reports comparing laparotomy versus laparoscopy for the surgical treatment of EC, and showed no significant difference (refer to Fig. 3). Galaal et al. conducted a Cochrane database analysis comparing nine randomized controlled trials (RCTs) that investigated laparoscopy and laparotomy for early-stage EC. Overall, this study presents low to moderate-certainty evidence supporting the use of laparoscopy in managing early EC, with similar OS and DFS rates [4].

**Table 3:** Surgical procedure, hospital stay and adjuvant therapies divided by time cohorts

	Total *N* (% valid)	1998–2000	2001–2005	2006–2010	2011–2014
*Surgical procedure*					
*N*	301	55	76	99	71
Non	2 (0.6)	0 (0)	1 (1.3)	0 (0)	1 (1.4)
Abdominal HE	245 (81.4)	49 (89.1)	70 (92.1)	95 (96)	31 (43.7)
TLH	36 (12)	0 (0)	0 (0)	0 (0)	36 (50.7)
Vaginal HE	18 (6)	6 (11)	5 (6.6)	4 (4)	3 (4.2)
*Lymphonodectomy (LNE)*					
*N*	300	55	75	98	72
Non	239 (79.7)	43 (78.2)	62 (82.7)	77 (78.6)	57 (79.2)
Pelvic	29 (9.7)	12 (21.8)	11 (14.7)	4 (4.1)	2 (2.8)
Pelvic + paraaortic	32 (10.7)	0 (0)	2 (2.7)	17 (17.3)	13 (18.1)
*Lymph nodes (LN)*					
*N* (with histology)	58	9	13	20	15
LN metastases of cases with LNE	15 (25.9)	1 (11.1)	3 (23.1)	5 (25.0)	6 (40)
Postop. stay (mean in days)	13.6	20.9	14.4	10.7	8.5
*Adjuvant therapies*					
Radiation	125	15	26	52	32
Chemotherapy	25	3	4	8	10
Endocrine	10	2	4	3	1

During the observed period the first TLH for EC and para-aortic LNE were performed at the institution. According to German S3 guidelines for the treatment of EC, systemic LNE is recommended for EC stage Ib and G3 or stage II and higher, regardless of grading, if total tumor resection can be achieved. A systemic LNE should include pelvic and infrarenal para-aortic LNE [2]. When comparing both techniques, it was found that a relatively lower number of lymph node (LN)metastases were present in the time cohorts when solely pelvic lymph node dissection (LND) was the standard procedure. Specifically, 11.1% of cases in 1998–2000 and 23.1% in 2001–2005 had LN metastases, compared to 25.0% in 2006–2010 and 37.5% in 2011–2014. However, this observation did not reach statistical significance (*p* = 0.509). It should be noted that the performance of LNE and the number of tumor-positive LNs were not correlated with tumor stage or histology. The present study does not aim to demonstrate the superiority of one technique over the other. The efficacy of LNE for EC has been evaluated in several randomized prospective trials. The MRC ASTEC trial found no evidence of a benefit regarding OS or DFS for LNE. The study population comprises solely of women with early-stage disease, specifically those with histologically proven EC that is believed to be confined to the corpus prior to surgery. The majority of the tumors were low risk, accounting for 49% in the non-LNE arm. In addition, the study only compared pelvic LNE to non-LNE [23]. The SEPAL retrospective cohort study has shown that combining para-aortic LNE with pelvic node dissection improves the survival of EC patients with postoperative intermediate or high risk of recurrence, but not for those with low risk of recurrence. Prospective randomized controlled studies addressing this question are of high clinical interest, and there are currently two ongoing clinical trials. The German ECLAT trial is currently enrolling patients with stage I and II EC who are at high risk of recurrence. The trial aims to investigate the effects of comprehensive pelvic and para-aortic LNE on patient outcomes. Results are expected in 2031 [24]. The Japan Clinical Oncology Group is enrolling the SEPAL-P3 study to compare pelvic and para-aortic LNE to pelvic LNE alone in patients with stage IB, II, IIIA, IIIB, and IIIC1 EC. The primary endpoint is OS. Results are pending [25].

The study has limitations due to its retrospective design, which means that confounding variables such as selection bias may have affected the results. Regarding the surgical approach, patient selection may have been biased against those with multimorbidity or obesity, which could have impacted the outcome after TAH. In addition, the number of performed VH was very low (*N* = 18, 6% of all HE), and therefore, this group was excluded from outcome calculations. The data were collected from the hospital’s electronic data processing system. Therefore, the follow-up period of patients varies, and we cannot rule out the possibility that recurrent disease or disease-associated deaths were not recorded. It is conceivable that patients consulted other hospitals in case of recurrence and were lost to follow-up for this study.

## Conclusions

Our single-center retrospective study demonstrates that pelviscopic surgery for EC is oncologically safe and reduces hospital stay duration. This is consistent with recently published data. Large multicenter RCTs are currently underway to investigate the prognostic impact of systemic LNE for EC patients.

## Data Availability

Data supporting the study results can be provided followed by request sent to the corresponding author’s e-mail.

## References

[1] Krebs in Deutschland für 2019/2020 (2023) Robert Koch-Institut und die Gesellschaft der epidemiologischen Krebsregister in Deutschland e.V

[2] S3-Leitlinie Endometriumkarzinom, Langversion 2.0 (2022) accessed 19.02.2024 https://www.leitlinienprogramm-onkologie.de/leitlinien/endometriumkarzinom/

[3] Acholonu UC (2012). Laparoscopy for the management of early-stage endometrial cancer: from experimental to standard of care. J Minim Invasive Gynecol.

[4] Galaal K et al (2018) Laparoscopy versus laparotomy for the management of early stage endometrial cancer. Cochrane Database Syst Rev 10(10):CD006655 10.1002/14651858.CD006655.pub3 PMC6517108 30379327

[5] Solmaz U (2016). Stage-III and -IV endometrial cancer: a single oncology centre review of 104 cases. J Obstet Gynaecol.

[6] Kim SI (2021). Minimally invasive surgery for patients with advanced stage endometrial cancer. Int J Med Sci.

[7] Tozzi R (2005). Laparoscopy versus laparotomy in endometrial cancer: first analysis of survival of a randomized prospective study. J Minim Invasive Gynecol.

[8] Reijntjes B (2022). Recurrence and survival after laparoscopy versus laparotomy without lymphadenectomy in early-stage endometrial cancer: long-term outcomes of a randomised trial. Gynecol Oncol.

[9] Tozzi R (2005). Analysis of morbidity in patients with endometrial cancer: is there a commitment to offer laparoscopy?. Gynecol Oncol.

[10] Walker JL (2012). Recurrence and survival after random assignment to laparoscopy versus laparotomy for comprehensive surgical staging of uterine cancer: gynecologic oncology group LAP2 study. J Clin Oncol.

[11] Zullo F, Falbo A, Palomba S (2012). Safety of laparoscopy vs laparotomy in the surgical staging of endometrial cancer: a systematic review and metaanalysis of randomized controlled trials. Am J Obstet Gynecol.

[12] Shepherd JH (1989). Revised FIGO staging for gynaecological cancer. Br J Obstet Gynaecol.

[13] Pecorelli S (2009). Revised FIGO staging for carcinoma of the vulva, cervix, and endometrium. Int J Gynaecol Obstet.

[14] Baum S (2022). Surgical treatment of endometrioid endometrial carcinoma—laparotomy versus laparoscopy. J Turk Ger Gynecol Assoc.

[15] Fram KM (2002). Laparoscopically assisted vaginal hysterectomy versus abdominal hysterectomy in stage I endometrial cancer. Int J Gynecol Cancer.

[16] Lu Q (2013). Comparison of laparoscopy and laparotomy for management of endometrial carcinoma: a prospective randomized study with 11-year experience. J Cancer Res Clin Oncol.

[17] Malzoni M (2009). Total laparoscopic hysterectomy versus abdominal hysterectomy with lymphadenectomy for early-stage endometrial cancer: a prospective randomized study. Gynecol Oncol.

[18] Zorlu CG, Simsek T, Ari ES (2005). Laparoscopy or laparotomy for the management of endometrial cancer. JSLS.

[19] Zullo F et al (2009) Laparoscopic surgery vs laparotomy for early stage endometrial cancer: long-term data of a randomized controlled trial. Am J Obstet Gynecol 200(3):296 e1–9 10.1016/j.ajog.2008.10.056 19167698

[20] Mourits MJ (2010). Safety of laparoscopy versus laparotomy in early-stage endometrial cancer: a randomised trial. Lancet Oncol.

[21] Gao H, Zhang Z (2015). Laparoscopy versus laparotomy in the treatment of high-risk endometrial cancer: a propensity score matching analysis. Medicine (Baltimore).

[22] OECD and E. Union (2020) Health at a glance: Europe 2020

[23] Kitchener H, ASTEC study group (2009). Efficacy of systematic pelvic lymphadenectomy in endometrial cancer (MRC ASTEC trial): a randomised study. Lancet.

[24] Emons G et al (2021) Endometrial Cancer Lymphadenectomy Trial (ECLAT) (pelvic and para-aortic lymphadenectomy in patients with stage I or II endometrial cancer with high risk of recurrence; AGO-OP.6). Int J Gynecol Cancer 31(7):1075–1079 10.1136/ijgc-2021-002703 34226291

[25] Watari H (2017). Phase III trial to confirm the superiority of pelvic and para-aortic lymphadenectomy to pelvic lymphadenectomy alone for endometrial cancer: Japan Clinical Oncology Group Study 1412 (SEPAL-P3). Jpn J Clin Oncol.

